# Tuberculose na infância e adolescência: prevalência e fatores associados ao abandono do tratamento

**DOI:** 10.1590/0102-311XPT158323

**Published:** 2024-09-16

**Authors:** Mariana Pereira da Soledade, Sueli Miyuki Yamauti, Andressa Simões Aguiar, Carolina Sucupira, Márcia Teresinha Lonardoni Crozatti

**Affiliations:** 1 Hospital Infantil Cândido Fontoura, São Paulo, Brasil.; 2 Universidade Federal de São Paulo, São Paulo, Brasil.; 3 Hospital Universitário da Universidade Federal de São Paulo, São Paulo, Brasil.; 4 Universidade de Sorocaba, Sorocaba, Brasil.; 5 Hospital São Luiz Gonzaga, São Paulo, Brasil.; 6 Hospital Infantil Darcy Vargas, São Paulo, Brasil.

**Keywords:** Tuberculose, Pediatria, Pacientes Desistentes do Tratamento, Fatores de Risco, Tuberculosis, Pediatrics, Patient Dropouts, Risk Factors, Tuberculosis, Pediatría, Pacientes Desistentes del Tratamiento, Factores de Riesgo

## Abstract

A tuberculose (TB) é uma doença infectocontagiosa que ainda representa um grave problema de saúde pública no mundo. Na população pediátrica, os fatores que levam ao abandono do tratamento da TB, especialmente em regiões de elevada prevalência da doença, são pouco conhecidos. Portanto, este estudo objetivou identificar a prevalência e os fatores de risco associados ao abandono do tratamento da TB em crianças e adolescentes. Foi realizado um estudo transversal com dados obtidos das notificações de TB provenientes do Sistema de Controle de Pacientes com Tuberculose do Estado de São Paulo, Brasil, em indivíduos com idade entre 0 e 18 anos, no período de janeiro de 2009 a dezembro de 2019. Estimou-se a razão de prevalência bruta e ajustada com intervalo de 95% de confiança, utilizando-se o modelo de regressão de Poisson para identificar associações entre o desfecho abandono do tratamento com os fatores sociodemográficos, clínico-epidemiológicos, diagnósticos e terapêuticos dos casos de TB, contendo informações completas. Dos 12.256 casos analisados, 941 indivíduos abandonaram o tratamento. A maior taxa de prevalência de abandono do tratamento ocorre entre os adolescentes pretos ou pardos, acima de 11 anos e privados de liberdade. Outras características associadas ao abandono do tratamento incluem: serem pessoas vivendo com HIV/aids, ter histórico de tratamento anterior para TB, fazer uso de substâncias ilícitas e utilizar o regime de tratamento de TB autoadministrado. Concluiu-se que conhecer o perfil do paciente com maiores chances para abandonar o tratamento da TB permite elaborar estratégias focadas na adesão ao tratamento medicamentoso mais efetivas.

## Abstract (en)

Tuberculosis (TB) is an infectious disease that remains a serious public health problem worldwide. In the pediatric population, the knowledge about the factors that lead to the abandonment of TB treatment is limited, especially in regions with a high prevalence of the disease. This study aimed to identify the prevalence and risk factors associated with TB treatment abandonment in children and adolescents. A cross-sectional study was carried out using data obtained from TB notifications from the São Paulo State Tuberculosis Patient Control System, Brazil, for individuals aged between 0 and 18 years, from January 2009 to December 2019. The crude and adjusted prevalence ratios were estimated with a 95% confidence interval, using the Poisson regression model to identify associations between the outcome of treatment abandonment and the sociodemographic, clinical-epidemiological, diagnostic and therapeutic factors of TB cases with complete information. Of the 12,256 cases analyzed, 941 individuals abandoned treatment. The highest prevalence rate of treatment abandonment occurs among Black or brown adolescents, those over 11 years of age and those deprived of their liberty. Other characteristics associated with treatment abandonment include: being a person living with HIV/AIDS, having a history of previous TB treatment, using illicit substances and using a self-administered TB treatment regimen. Knowing the profile of the patient most likely to abandon TB treatment makes it possible to devise more effective strategies focused on adherence to drug treatment.

## Abstract (es)

La tuberculosis (TB) es una enfermedad infectocontagiosa que sigue siendo un grave problema de salud pública mundial. Se sabe poco sobre los factores que conducen al abandono del tratamiento de la TB en la población pediátrica, especialmente en regiones con alta prevalencia de la enfermedad. Por lo tanto, este estudio tuvo como objetivo identificar la prevalencia y los factores de riesgo asociados con el abandono del tratamiento de la TB en niños y adolescentes. Se trató de un estudio transversal realizado a partir de los datos de notificaciones de TB del Sistema de Control de Pacientes con Tuberculosis del Estado de São Paulo, Brasil, recopilados de individuos con edades comprendidas entre 0 y 18 años, en el período de enero de 2009 a diciembre de 2019. La relación de prevalencia bruta y ajustada se estimó con un intervalo de 95% de confianza; y se utilizó el modelo de regresión de Poisson para identificar las asociaciones entre el resultado del abandono del tratamiento y los factores sociodemográficos, clínico-epidemiológicos, diagnósticos y terapéuticos de los casos de TB que contienen información completa. De los 12.256 casos analizados, 941 individuos habían abandonado el tratamiento. La mayor tasa de prevalencia de abandono del tratamiento se dio entre adolescentes negros o pardos, mayores de 11 años y los privados de libertad. Otras características asociadas con el abandono del tratamiento fueron personas que viven con VIH/sida, tener antecedentes de tratamiento previo de TB, usar sustancias ilícitas y utilizar el régimen de tratamiento de TB autoadministrado. Se concluyó que conocer el perfil de los pacientes con mayor tendencia a abandonar el tratamiento de la TB nos permite desarrollar estrategias efectivas enfocadas en una adherencia al tratamiento farmacológico.

## Introdução

Segundo o *Boletim Global da Tuberculose* da Organização Mundial da Saúde (OMS) ^1^, estima-se que em 2022 cerca de 10,6 milhões de pessoas desenvolveram tuberculose (TB) no mundo. Dentre esses casos, 1,3 milhão (12%) ocorreram em crianças menores de 15 anos, levando a óbito aproximadamente 16% desses indivíduos, evidenciando um preocupante impacto da doença nesta população.

Apesar do reconhecimento de que a TB representa um grave problema de saúde pública global, apenas nas últimas décadas essa epidemia na população pediátrica, cuja real proporção não é totalmente conhecida, passou a receber a atenção necessária para prevenção e cuidado ^2^ ^,^ ^3^.

Neste contexto, é consenso que há uma menor disponibilidade de estudos sobre a TB em crianças e adolescentes quando comparado à população jovem e adulta, cuja incidência é mais elevada. Em relação às crianças e adolescentes, de maneira geral, essa defasagem de estudos parece estar ligada à dificuldade de identificação e de realização do diagnóstico diferencial da doença nessa faixa etária, principalmente se houver coinfecção com o HIV ou com a doença decorrente dessa infecção, a aids, fato que leva à subnotificação de casos e a dados epidemiológicos subestimados ^4^ ^,^ ^5^.

Dentre os estudos sobre TB nessa população, são menos frequentes aqueles que abordam os fatores que levam ao abandono e problemas de adesão ao tratamento, especialmente em regiões com elevada prevalência da doença ^6^. Alguns pesquisadores mencionam que a falta de uniformidade nas definições de adesão ao tratamento resulta em desafios ao sumarizar evidências e comparar resultados ^7^, limitando os estudos nesse campo. Adicionalmente, a definição de criança e adolescente, bem como seus limites etários, também divergem na literatura, dificultando a análise e a comparação dos dados por estratificação etária ^8^.

Mesmo diante das limitações, os estudos disponíveis enfatizam a necessidade de atenção a esse tema, haja vista as taxas significativas de abandono e falta de adesão ao tratamento, sobretudo entre adolescentes e jovens adultos do sexo masculino, conforme apontado em alguns estudos ^9^ ^,^ ^10^ ^,^ ^11^. Vale ressaltar, também, que os desafios relacionados à falta de adesão e ao abandono do tratamento da TB resultam na contínua propagação da doença, especialmente considerando a predominância da forma pulmonar entre os adolescentes ^9^. Esse quadro se associa a desfechos adversos, incluindo óbito, insucesso terapêutico e desenvolvimento de resistência ao tratamento ^11^.

Visando o desenvolvimento de um modelo conceitual para estudo do tema, Leddy et al. ^7^ categorizam os determinantes sociais que afetam a adesão e que podem levar crianças, adolescentes e jovens adultos ao abandono do tratamento de TB. De acordo com o modelo proposto, esses fatores podem ser categorizados como estruturais e/ou relacionados à comunidade, sistemas de saúde, domiciliares e individuais, evidenciando a complexidade da questão.

De fato, os fatores de risco para o abandono do tratamento de TB mais discutidos na literatura são: ser imigrante ou ter um genitor migrante/imigrante, desconhecimento sobre a doença e seu tratamento, estigma/discriminação relacionados à TB, distância entre a residência e os serviços de saúde, sensação de melhora dos sintomas, surgimento de efeitos colaterais, presença de comorbidades como infecção pelo HIV, má nutrição e abuso de álcool e de substâncias psicoativas ^7^ ^,^ ^8^ ^,^ ^12^. Pensando ainda que crianças e adolescentes dependem de um cuidador ou responsável legal para que seu tratamento seja concluído e/ou estimulado, a dinâmica familiar em que o paciente com TB está inserido também impactará no desfecho do tratamento ^13^.

Partindo do exposto, este estudo teve como objetivo identificar a prevalência e os fatores de risco associados ao abandono do tratamento de TB entre os casos notificados no Estado de São Paulo, Brasil, no período de 2009 a 2019.

## Método

Trata-se de um estudo transversal dos casos de TB em crianças e adolescentes notificados no período de janeiro de 2009 a dezembro de 2019, no Estado de São Paulo.

Os dados foram obtidos do Sistema de Controle de Pacientes com Tuberculose do Estado de São Paulo, o TBWeb (http://www.cvetb.saude.sp.gov.br/tbweb/). Esse sistema é alimentado pelas notificações e todo o acompanhamento dos casos de TB realizados em todas as instituições de saúde do estado e gerenciado pela Divisão de Tuberculose do Centro de Vigilância Epidemiológica “Prof Alexandre Vranjac” (CVE).

A base de dados foi disponibilizada anonimizada, pelo gestor do banco de dados à pesquisadora principal, em planilhas de Excel, versão 2011 (https://products.office.com/), contendo os dados da Ficha de Notificação de Tuberculose junto com os dados do seu acompanhamento. O acesso a essas planilhas ocorreu após a aprovação do projeto de estudo pelo Comitê de Ética em Pesquisa da Universidade Federal de São Paulo (UNIFESP; parecer nº 4.507.493) e pelo CVE.

Nesse estudo, foram incluídas todas as notificações encerradas de TB, segundo os critérios definidos pelo Ministério da Saúde ^14^, que foram diagnosticadas em qualquer órgão ou sistema, ocorridas em pacientes de 0 a 18 anos.

Foram excluídas da análise as notificações que apresentavam informações conflitantes ou duvidosas, assim como aquelas que apresentavam critérios de encerramento que não poderiam ser consideradas como abandono de tratamento, tais como: óbito, mudança de diagnóstico, falência de tratamento ou transferência do paciente do local de tratamento para outro estado ou país. As variáveis que não possuíam informações relevantes para o estudo também foram excluídas, bem como os dados indicados como ignorados.

Após esta etapa, as seguintes categorias de variáveis foram elencadas:

(1) Sociodemográficas: faixa etária, cor da pele/raça (branco, preto/pardo e outras cores/raças), sexo e região metropolitana de residência;

(2) Clínico-epidemiológicas: agravos associados (pessoa vivendo com HIV/aids e usuário de substâncias psicoativas) e local/forma de descoberta da doença;

(3) Diagnósticas e terapêuticas: tratamento anterior, esquema de tratamento inicial e regime de tratamento.

Foram calculadas as frequências absolutas e relativas do desfecho “abandono de tratamento” para cada uma das variáveis selecionadas, em suas respectivas categorias. As diferenças entre as proporções das variáveis nos grupos com e sem abandono foram estimadas por meio do teste χ^2^ de Pearson, considerando-se o nível de significância estatística de 5%.

A associação entre o desfecho “abandono do tratamento” e os fatores sociodemográficos, clínico-epidemiológicos, diagnósticos e terapêuticos foi estimada pela medida de associação entre a razão de prevalência (RP) bruta e ajustada, e seus respectivos intervalos de 95% de confiança (IC95%). A análise multivariável foi efetuada por meio do modelo de Poisson corrigido por variância robusta ^15^. O modelo multivariável final foi construído pela estratégia *stepwise forward selection*, sendo que a inclusão das variáveis levou em conta a estimativa da medida de associação e a significância estatística (p < 0,05) das variáveis na análise bivariada. Permaneceram no modelo final aquelas variáveis consideradas estatisticamente significantes (teste de wald; p < 0,05). Durante a modelagem, as variáveis foram avaliadas quanto à colinearidade, pelo teste de Spearman. A análise foi realizada usando o programa Stata (https://www.stata.com).

A associação entre a variável de residência na Região Metropolitana e o desfecho “abandono de tratamento” foi calculada considerando como expostos os indivíduos que residiam na Região Metropolitana de interesse, enquanto os não expostos compreendiam todos aqueles que não residiam na referida região. Essa escolha na apresentação dos resultados foi feita para viabilizar o cálculo do risco de abandono do tratamento para os pacientes residentes naquela Região Metropolitana em comparação com os residentes nas demais Regiões Metropolitanas. Dessa maneira, podemos ter uma ideia de como os determinantes sociais do abandono do tratamento de TB naquela região estão afetando os pacientes diagnosticados.

## Resultados

No período de estudo, foram notificados 12.872 casos de TB entre crianças e adolescentes. Após a exclusão de 616 notificações, conforme os critérios pré-estabelecidos, 12.256 casos foram analisados, dos quais 941 (7%) abandonaram o tratamento.

A maioria dos casos de abandono ocorreu entre adolescentes maiores de 11 anos (n = 755; 9,1%) e entre os indivíduos de cor da pele/raça preta/parda ou outras (n = 631; 8,8%), havendo ligeira maioria de casos de abandono dentre os indivíduos do sexo masculino (n = 554; 8,7%) (Tabela 1).

Ainda na Tabela 1, verifica-se que os casos com algum agravo associado (infecção pelo HIV ou uso de substância) ou histórico de tratamento de TB anterior apresentaram maiores taxas de abandono. Os pacientes com coinfecção tuberculose + HIV/aids apresentaram taxa semelhante de abandono, que ocorreu em 16,8% (n = 53) e 17,3% (n = 51) dos casos, respectivamente. Vale destacar que 33,4% (n = 210) dos usuários de substâncias psicoativas e 24% (n = 126) dos casos com tratamento anterior abandonaram o tratamento.

**Tabela 1:** Frequência de abandono do tratamento de tuberculose segundo as características sociodemográficas, clínico-epidemiológicas e terapêuticas, dos casos notificados em indivíduos de 0 a 18 anos, no Estado de São Paulo, Brasil, no período de 2009 a 2019.

Características	Abandono				Total	Valor de p
	Não		Sim			
	n	%	n	%		
Sociodemográficas						
Faixa etária (anos)						< 0,001
0-10	3.534	95,5	166	4,5	3.700	
11-18	7.781	90,9	755	9,1	8.556	
Cor da pele/Raça						< 0,001
Branco	4.788	93,2	310	6,1	5.098	
Pretos/Pardos e outros	6.527	91,2	631	8,8	7.158	
Sexo						< 0,001
Feminino	5.477	93,4	387	6,6	5.864	
Masculino	5.838	91,3	554	8,7	6.392	
Região Metropolitana de residência						
São Paulo						0,005
Não	4.457	93,2	327	6,8	4.784	
Sim	6.858	91,8	614	8,2	7.472	
Baixada Santista						0,144
Não	9.959	92,4	813	7,5	10.772	
Sim	1.356	91,4	128	8,6	1.484	
Campinas						< 0,001
Não	10.672	92,1	914	7,9	11.586	
Sim	643	96	27	4,0	670	
Ribeirão Preto						0,130
Não	11.062	92,3	927	7,7	11.989	
Sim	253	94,8	14	5,2	267	
Sorocaba						0,846
Não	11.039	92,3	919	7,7	11.958	
Sim	404	95,6	21	4,9	425	
Vale do Paraíba e Litoral Norte						0,031
Não	10.911	92,2	920	7,8	11.831	
Sim	404	95,6	21	4,9	425	
Municípios não metropolitanos						< 0,001
Não	9.891	91,9	875	8,1	10.766	
Sim	1.424	95,6	66	4,4	1.490	
População privada de liberdade						< 0,001
Não	11.214	92,6	892	7,4	12.106	
Sim	101	67,3	49	32,7	150	
Clínicas, epidemiológicas e terapêuticas *						
Agravos associados						
Pessoa vivendo com aids **						< 0,001
Não	11.045	92,6	888	7,4	11.933	
Sim	244	82,7	51	17,3	295	
Usuário de substâncias psicoativas ***						< 0,001
Não	10.892	93,7	731	6,3	11.623	
Sim	419	66,6	210	33,4	629	
Pessoa vivendo com HIV ^#^						< 0,001
Não	9.054	93,8	596	6,2	9.650	
Sim	262	83,2	53	16,8	315	
Tratamento inicial						< 0,001
RHZ	3.093	94,4	184	5,6	3.277	
RHZE	7.988	91,8	711	8,2	8.699	
Outros	232	87,5	33	12,4	265	
Regime de tratamento						< 0,001
Supervisionado	8.091	93,8	527	6,1	8.618	
Autoadministrado	2.518	89,3	300	10,6	2.818	
Histórico de tratamento anterior						< 0,001
Não	10.917	93,0	815	6,9	11.732	
Sim	398	75,9	126	24,0	524	
Locais/Formas de descoberta						
Pronto socorro						< 0,001
Não	8.954	92,8	691	7,2	9.645	
Sim	2.137	90,2	232	9,8	2.369	
Ambulatório						0,213
Não	6.637	92,6	533	7,4	7.170	
Sim	4.454	91,9	390	8,0	4.844	
Busca ativa						0,001
Não	10.085	92,4	844	7,5	11.709	
Sim	266	87,2	39	12,8	305	
Internado						0,270
Não	8.869	92,2	752	7,8	9.621	
Sim	2.222	92,8	171	7,1	2.393	
Investigação de contatos						< 0,001
Não	9.079	91,6	832	8,4	9.911	
Sim	2.012	95,6	91	4,3	2.103	

RHZ: rifampicina, isoniazida e pirazinamida; RHZE: rifampicina, isoniazida, pirazinamida e etambutol.

Nota: foi considerado estatisticamente significativo p < 0,05.

* Nas variáveis de agravos associados e locais/formas de descoberta, foram desconsiderados os casos com a informação do agravo ignorado ou de exame não coletado;

** Refere-se ao termo “aids” utilizado no banco de dados original;

*** Refere-se ao termo “drogadição” utilizado no banco de dados original;

^#^ Refere-se ao termo “sorologia positiva para HIV” utilizado no banco original.

Em relação aos locais ou formas de diagnóstico da TB, os casos diagnosticados por meio de busca ativa possuem a maior taxa de abandono (n = 39; 12,8%), seguidos dos casos diagnosticados no pronto socorro (n = 232; 9,8%) e os diagnosticados por meio de investigação de contatos (n = 91; 4,3%). Já os casos diagnosticados no ambulatório (n = 390; 8%) e durante internação (n = 171; 7,1%) não apresentam diferença significativa (p > 0,05) quando comparados com aqueles que abandonaram o tratamento e não foram diagnosticados nesses ambientes (Tabela 1).

Quanto ao esquema de tratamento inicial, os casos que utilizaram rifampicina, isoniazida, pirazinamida e etambutol (RHZE) e outros esquemas foram os que mais o abandonaram, 8,2% (n = 711) e 12,4% (n = 33), respectivamente (Tabela 1).

Dentre as Regiões Metropolitanas de residência dos casos notificados, a de São Paulo (capital) foi a que apresentou maior taxa de abandono (n = 614; 8,2%), seguida do Vale do Paraíba e Litoral Norte (n = 21; 4,9%) e Campinas (n = 27; 4%). Para os casos que residiam em regiões não metropolitanas, a taxa de abandono foi de 4,4% (n = 66). A população privada de liberdade apresentou taxa de abandono de 32,7% (n = 49) (Tabela 1).

A Tabela 2 apresenta as RPs bruta e ajustada das características sociodemográficas e clínico-epidemiológicas que podem estar associadas ao abandono de tratamento de TB. Após análise multivariada, verificou-se que os casos que estão na faixa etária 11-18 anos (RP = 1,75; IC95%: 1,36-2,25) com pacientes pretos/pardos ou com outras cores de pele (RP = 1,32; IC95%: 1,12-1,55) ou estão em condição de privação de liberdade (RP = 1,90; IC95%: 1,36-2,67) apresentaram probabilidade de abandono do tratamento aproximadamente 1,5 vez maior. Ser uma pessoa vivendo com HIV, histórico de tratamento de TB anterior ou uso de substâncias psicoativas foram características que se apresentaram, estatisticamente, associados ao aumento da prevalência para abandono, a qual pode chegar a ser até quase cinco vezes maior, caso, por exemplo, o paciente seja usuário de substâncias psicoativas (RP = 4,56; IC95%: 3,79-5,48). O tratamento autoadministrado também mostrou-se como fator associado com abandono (RP = 1,84; IC95%: 1,56-2,17). Por fim, residir em um município não metropolitano se apresentou como fator de proteção ao abandono de tratamento de TB (RP = 0,61; IC95%: 0,45-0,83).

**Tabela 2:** Estimativa das razões de prevalência (RP) bruta e ajustada pelo modelo de regressão de Poisson das características sociodemográficas, clínico-epidemiológicas e terapêuticas associadas ao abandono do tratamento de tuberculose, dos casos notificados em indivíduos de 0 a 18 anos, no Estado de São Paulo, Brasil, no período de 2009 a 2019.

Características	RP bruta	IC95%	Valor de p	RP ajustada	IC95%	Valor de p
Sociodemográficas						
Faixa etária (anos)						
0-10	1,00					
11-18	2,02	1,71-2,38	0,000	1,75	1,36-2,25	< 0,001
Cor da pele/Raça						
Branco	1,00					
Pretos/Pardos e outros	1,45	1,27-1,65	0,000	1,32	1,12-1,55	< 0,001
Sexo						
Feminino	1,00					
Masculino	1,31	1,16-1,49	0,000	1,07	0,91-1,21	0,382
Região Metropolitana de residência						
São Paulo						
Não	1,00					
Sim	1,2	1,05-1,37	0,005	1,14	0,96-1,36	0,130
Baixada Santista						
Não	1,00					
Sim	1,14	0,96-1,36	0,144	-	-	-
Campinas						
Não	1,00					
Sim	0,51	0,35-0,74	0,000	0,77	0,48-1,26	0,309
Ribeirão Preto						
Não	1,00					
Sim	0,68	0,40-1,13	0,130	-	-	-
Sorocaba						
Não	1,00					
Sim	0,96	0,64-1,44	0,846	-	-	-
Vale do Paraíba e Litoral Norte						
Não	1,00					
Sim	0,63	0,42-0,97	0,031	0,86	0,52-1,43	0,569
Municípios não metropolitanos						
Não	1,00					
Sim	0,54	0,43-0,69	0,000	0,61	0,45-0,83	0,002
População privada de liberdade						
Não	1,00					
Sim	4,43	3,49-5,62	0,000	1,90	1,36-2,67	< 0,001
Clínicas, epidemiológicas e terapêuticas						
Agravos associados						
Pessoa vivendo com aids *						
Não	1,00					
Sim	2,32	1,79-3,00	0,000	1,25	0,59-2,66	0,556
Usuário de substâncias psicoativas **						
Não	1,00					
Sim	5,31	4,66-6,05	0,000	4,56	3,79-5,48	< 0,001
Pessoa vivendo com HIV ***						
Não	1,00					
Sim	2,72	2,10-3,52	0,000	1,81	1,37-2,39	< 0,001
Tratamento inicial						
RHZ	1,00					
RHZE	1,33	1,15-1,54	0,000	0,89	0,70-1,13	0,338
Outros	1,67	1,20-2,31	0,002	1,03	0,64-1,68	0,887
Regime de tratamento						
Diretamente observado	1,00					
Autoadministrado	1,74	1,52-1,99	0,000	1,84	1,56-2,17	< 0,001
Histórico de tratamento anterior						
Não	1,00					
Sim	3,46	2,93-4,09	0,000	2,53	2,04-3,12	< 0,001
Locais/Formas de descoberta						
Pronto socorro						
Não	1,00					
Sim	1,37	1,18-1,57	0,000	1,05	0,88-1,26	0,579
Ambulatório						
Não	1,00					
Sim	1,08	0,95-1,23	0,213	-	-	-
Busca ativa						
Não	1,00					
Sim	1,69	1,25-2,28	0,001	1,11	0,74-1,67	0,606
Internado						
Não	1,00					
Sim	0,92	0,80-1,06	0,270	-	-	-
Investigação de contatos						
Não	1,00					
Sim	0,51	0,42-0,66	0,000	0,79	0,58-1,08	0,145

IC95%: intervalo de 95% de confiança; RHZ: rifampicina, isoniazida e pirazinamida; RHZE: rifampicina, isoniazida, pirazinamida e etambutol.

Nota: foi considerado estatisticamente significativo p < 0,05.

* Refere-se ao termo “aids” utilizado no banco de dados original;

** Refere-se ao termo “drogadição” utilizado no banco de dados original;

*** Refere-se ao termo “sorologia positiva para HIV” utilizado no banco original.

## Discussão

Observando os dados da Tabela 1, verifica-se que a taxa de abandono encontrada é superior ao preconizado como aceitável pela OMS (< 5%) para o controle da TB ^16^. Entretanto, em revisão da literatura, verificou-se que a taxa encontrada foi muito semelhante à apresentada por Galli et al. ^4^ em sua análise de dados de TB em crianças italianas. Os autores encontraram uma frequência de 8% dos casos diagnosticados. Já o estudo de Kebede et al. ^17^ com menores de 15 anos do noroeste da Etiópia encontrou uma taxa de abandono de 21,1%, muito superior à do estudo realizado. Taxas de abandono inferiores foram encontradas por Hailu et al. ^18^ em estudo retrospectivo de cinco anos dos dados de TB em menores de 15 anos dos centros de saúde da capital da Etiópia; e em Siamisang et al. ^5^, em estudo retrospectivo realizado em Botswana. Conforme esses autores, cerca de 3,8% e 3,4% dos casos estudados abandonaram o tratamento, respectivamente.

Acredita-se que a diferença observada entre as taxas de abandono do tratamento de TB em diferentes regiões do mundo pode estar associada à realidade em que o paciente está inserido. Haja vista as características da doença, cuja expansão se dá em situações de maior vulnerabilidade clínica e social, o tratamento desta está sujeito, também, à ação de vários determinantes sociais. Dessa forma, é preciso que a prevenção ao abandono do tratamento de TB ocorra por meio de estratégias que estejam em conformidade com o perfil do paciente que apresenta maiores probabilidades de abandono ^19^. Esse perfil, entretanto, pode variar entre populações e regiões, sendo necessário conhecê-lo para que intervenções sejam realizadas.

Tendo em vista, portanto, o desenho do perfil dos casos notificados no Estado de São Paulo, verificou-se que a maioria dos casos de abandono ocorreu entre indivíduos a partir dos 11 anos, com probabilidade de abandono quase duas vezes maior entre os mais velhos. Enane et al. ^20^ mostraram que adolescentes apresentam risco de abandono do tratamento de TB duas vezes maiores do que adultos (RR = 2,0; IC95%: 1,1-3,7; p = 0,03), especialmente se houver coinfecção TB/HIV. Essa elevação dos índices de abandono do tratamento dentre os indivíduos mais velhos era esperada, pois a adolescência é caracterizada por um período turbulento, marcado por mudanças físicas, psicológicas e comportamentais ^21^, o qual pode tornar-se mais acentuado a depender do ambiente social em que o indivíduo vive. Sendo assim, ações estratégicas para essa população devem ser pensadas considerando suas peculiaridades.

Ainda em relação às características sociodemográficas da população que compunha o banco de dados, observou-se que o grupo dos indivíduos pretos/pardos e de outras cores/raças apresentaram maior probabilidade de abandono do tratamento do que os indivíduos que se consideram brancos. Esse fato pode estar associado aos determinantes sociais que atingem de forma desigual a população brasileira, tornando parte dela mais vulnerável ao desenvolvimento da TB e ao abandono do tratamento. No Brasil, as classes sociais com menor renda familiar são formadas, em sua maioria, por grupos populacionais de cor/raça preta e parda. Em 2021, cerca de 34% da população preta e 38% da população parda viviam abaixo da linha da pobreza ^22^. De acordo com Navarro et al. ^23^, a renda familiar está associada à maior chance de abandono de tratamento de TB, devendo o Estado agir para promover a equidade em seus programas de promoção e proteção à saúde. Assim, a classe social em que a criança ou adolescente está inserida pode ser um fator de impacto no abandono do tratamento.

Pacientes em privação de liberdade, grupo formado por indivíduos com idade superior ou igual a 12 anos, apresentaram probabilidade de abandono superior em relação à população geral. Segundo dados da Fundação Casa ^24^, cerca de 91,4% de seus internos são adolescentes a partir de 15 anos, sendo 95% do sexo masculino e 70,1% de pretos ou pardos. Dessa maneira, essa população reúne características dos grupos já citados anteriormente que se revelaram propensos ao abandono, segundo o modelo de análise utilizado, além de possuir características próprias relativas ao comportamento de risco e vulnerabilidade social. Como o tratamento da TB é longo e pode superar o período de internação na Fundação Casa, em que o tratamento é supervisionado, verifica-se tendência à falha de adesão após a saída do sistema socioeducativo. Esse dado mostra a necessidade de construção de um sistema de compartilhamento das informações dos pacientes em tratamento para TB, provenientes do sistema socioeducativo com os serviços da atenção básica de saúde, permitindo o acolhimento e o vínculo desse adolescente e sua família com o serviço de saúde. Dessa forma, assegura-se a continuidade do tratamento, tal qual determinam os princípios e diretrizes gerais da atenção básica, que de acordo com a Política Nacional da Atenção Básica ^25^ orientam-se, dentre outros, pelos princípios da universalidade, vínculo, continuidade do cuidado, integralidade da atenção e humanização. Em estudo realizado em Belo Horizonte (Minas Gerais) ^26^, que objetivou avaliar o desempenho da atenção primária no controle da TB, os autores averiguaram que o sistema de contrarreferência de informações clínicas dos pacientes em tratamento de TB não funcionava adequadamente. Esse problema, segundo os autores, é compartilhado por estudos semelhantes, sugerindo que a falta de integração de informações por meio de sistema informatizado afeta não somente o controle da TB, mas também de outras patologias que precisam ser enfrentadas com urgência.

Em relação às características clínicas dos casos notificados, verificou-se, assim como demonstrado por outros autores ^27^, que ser uma pessoa que vive com HIV, usuária de substância ilícitas e possuir histórico de tratamento anterior para TB elevaram a probabilidade de abandono. Dentre essas características, o uso de substâncias psicoativas ficou em evidência, pois elevou a probabilidade de abandono em até cinco vezes, sendo a característica que apresentou maior probabilidade de abandono, seguida do histórico de tratamento anterior.

Quanto ao regime de tratamento realizado, os dados deste trabalho mostraram que a estratégia de tratamento diretamente observado (TDO) é superior para garantir a finalização do tratamento. A função dessa estratégia é justamente monitorar e garantir a adesão ao tratamento ^28^. Sabendo-se que muitos dos casos de abandono possuem características de risco somadas, a realização do tratamento autoadministrado representa perda da oportunidade de identificação dos preditores de abandono preexistentes, devido à falta da monitorização contínua do tratamento e do estabelecimento de vínculo com o paciente. Por meio do vínculo formado entre profissionais e pacientes no TDO, os grupos socialmente vulneráveis, caracterizados pela dificuldade de adesão ao tratamento, pelo baixo acesso aos serviços de saúde e maior incidência de desfechos desfavoráveis, são melhor monitorados ^29^.

Por fim, a única característica dentro do modelo de análise utilizado neste estudo que se apresentou como fator protetor ao abandono foi a de residência em municípios não metropolitanos. Apesar de parecer controverso, já que as áreas metropolitanas e áreas altamente urbanizadas apresentam maior oferta de serviços de saúde e infraestrutura, as capitais brasileiras apresentam as maiores taxas de abandono ^16^. Alguns autores afirmam que devido à maior heterogeneidade territorial, áreas mais densamente povoadas têm maior dificuldade na criação de vínculo com suas comunidades em comparação com localidades menos povoadas ^30^. Pensando, portanto, que as Regiões Metropolitanas agregam as maiores densidades populacionais do estado, os dados encontrados corroboram os fatos citados.

Embora na amostra utilizada haja pacientes incapazes de realizar o autocuidado devido à faixa etária, não estavam disponíveis informações a respeito do cuidador responsável pela administração do medicamento desses pacientes. Desse modo, características específicas desses cuidadores que possam ser preditoras de risco para abandono do tratamento dos pacientes sob seus cuidados não foram possíveis de serem identificadas.

## Conclusão

A taxa de abandono do tratamento da TB encontrada neste estudo é superior ao estipulado pela OMS como aceitável. No entanto, não difere do observado em algumas partes do mundo. De fato, o perfil do paciente com maior predisposição para o abandono do tratamento abrange aqueles com maior vulnerabilidade por suas características sociodemográficas, comportamentais e histórico clínico-epidemiológico. O estudo identificou que os pacientes entre 11 e 18 anos, pretos/pardos ou outras cores/raças, vivendo com o HIV, usuários de substâncias psicoativas, com histórico de tratamento anterior para TB, em tratamento auto administrado e privação de liberdade possuem maior probabilidade de abandonar o tratamento da TB, podendo chegar a ser cinco vezes maior, quando comparados com aqueles que não possuem as características mencionadas.

Dentro do modelo de análise, a única característica que se mostrou protetora ao abandono foi a residência em municípios não metropolitanos, possivelmente devido à capacidade dos serviços de áreas menos populosas de estabelecer maior vínculo com seus pacientes. Dadas as peculiaridades do tratamento da TB, o vínculo estabelecido entre paciente/cuidadores e os profissionais de saúde mostra-se fundamental para desfechos de sucesso.

Acredita-se que este estudo possa contribuir no desenho de um perfil de risco para o abandono do tratamento de TB. Assim, estratégias preventivas podem ser traçadas com maior eficácia.

Destaca-se a necessidade do fortalecimento do vínculo entre profissionais da saúde e pacientes na atenção primária da consolidação do TDO como padrão ouro no manejo da TB; e a necessidade da implantação de um sistema informatizado de informações clínicas integrado entre o sistema penitenciário e o Sistema Único de Saúde e seus diversos níveis de atenção à saúde.
